# Multi-Stage Meta-Learning for Few-Shot with Lie Group Network Constraint

**DOI:** 10.3390/e22060625

**Published:** 2020-06-05

**Authors:** Fang Dong, Li Liu, Fanzhang Li

**Affiliations:** School of Computer Science and Technology, Soochow University, Suzhou 215006, China; 20185227041@stu.suda.edu.cn (F.D.); lliu6819@suda.edu.cn (L.L.)

**Keywords:** meta-learning, lie group, machine learning, deep learning, convolutional neural network

## Abstract

Deep learning has achieved many successes in different fields but can sometimes encounter an overfitting problem when there are insufficient amounts of labeled samples. In solving the problem of learning with limited training data, meta-learning is proposed to remember some common knowledge by leveraging a large number of similar few-shot tasks and learning how to adapt a base-learner to a new task for which only a few labeled samples are available. Current meta-learning approaches typically uses Shallow Neural Networks (SNNs) to avoid overfitting, thus wasting much information in adapting to a new task. Moreover, the Euclidean space-based gradient descent in existing meta-learning approaches always lead to an inaccurate update of meta-learners, which poses a challenge to meta-learning models in extracting features from samples and updating network parameters. In this paper, we propose a novel meta-learning model called Multi-Stage Meta-Learning (MSML) to post the bottleneck during the adapting process. The proposed method constrains a network to Stiefel manifold so that a meta-learner could perform a more stable gradient descent in limited steps so that the adapting process can be accelerated. An experiment on the mini-ImageNet demonstrates that the proposed method reached a better accuracy under 5-way 1-shot and 5-way 5-shot conditions.

## 1. Introduction

Traditional deep learning models are dedicated to extracting embedding features from large scale data that belong to the same distribution and use them to classify new samples. With the fast growth of computing capacity and the scale of labeled samples, deep learning has achieved great performances, especially in image classification and regression prediction, some of them even with better performances than humans in many scenes [1]. The success of deep learning generally depends on fast-enough computers and enough data to actually train large neural networks. Most deep learning models could benefit from performance improvements as the number of available training data increases. However, in some cases where there are insufficient amounts of labeled samples, a deep learning model could result in an overfitting problem. Machine learning society expects a solution to design a deep learning model with similar properties; learning new concepts and skills fast with a few training examples.

Meta-learning [2,3] has been proposed as a framework to address the challenging problems of insufficient training data and rapid task adaption requirements that lies in many machine learning models. The basic idea here is to leverage a large number of similar few-shot tasks in order to learn how to adapt a base-learner to a new task for which only a few labeled samples are available. In contrast to data-augmentation methods, meta-learning is a task-level learning method. Meta-learning aims to accumulate experience from learning multiple tasks, while base-learning focuses on modeling the data distribution of a single task. Current research on meta-learning can be broadly categorized into three classes: (1) A metric-based approach that first calculates the similarity between samples then classifies these samples into predicted results by a measure of distance like cosine [4,5,6]; (2) memory-based approach that uses a small extra neural network to expand the learning capacity of a meta-learner [7,8]; and (3) gradient decent-based approach in which every layer of the neural network inside a meta-learner is updated by applying gradient descent [9,10,11]. Despite the difference between these approaches, current research typically uses Shallow Neural Networks (SNNs) to avoid overfitting, thus wasting much information in adapting to a new task. Moreover, Euclidean space-based gradient descent always leads to an inaccurate update of meta-learners, which poses a challenge to meta-learning models in extracting features from samples and updating network parameters.

In this paper, we propose a new method called Multi-Stage Meta-Learning for Few-Shot with Lie Group Network Constraint to improve the performance of a meta-learning model in adapting to a new task and updating the meta-learner. Our method is based on Model-Agnostic Meta-Learning (MAML) [9], a representative gradient decent-based approach that is considered to provide an all-round good performance. Compared to the existing MAML methods, the proposed method has three major advantages:

(1) Reducing the number of parameters that needs to be updated during meta-train and meta-test phases by using a deeper network structure in which a pretrain operation on meta-learner’s low-level layers is performed;
(2) Improving the utilization level of training data with limited updating steps by introducing a multi-stage optimization mechanism in which the model sets some checkpoints during the middle and late stage in the process of adaption;
(3) Constraining a network to Stiefel manifold so that a meta-learner could perform a more stable gradient descent in limited steps and accelerate the adapting process.

An experiment on the mini-ImageNet [4] demonstrated that the proposed method reaches a better accuracy under 5-way 1-shot and 5-way 5-shot conditions.

The remainder of this paper is organized as follows: Section 2 reviews the related work in meta-learning and Section 3 introduces the proposed method in detail. The experiment results on an open source image dataset mini-ImageNet are reported in Section 4. In Section 5, the experiment results along with the effectiveness of the proposed method are discussed. Finally, Section 6 summarizes this work and Section 7 discusses potential future work.

## 2. Related Work

Previous research related to the methodologies adopted in this paper includes MAML-based meta-learning models and gradient descent optimization in meta-learning models.

### 2.1. MAML-Based Meta-Learning Models

As a gradient decent-based approach, MAML learns a generalized initial network with a great compatibility of different updating rules and networks. A MAML-based model includes three components: (a) Common initial basics; (b) a process of adaption; and (c) a set of gradient-based rules to update network parameters inside meta-learner. These three parts correspond to the basic knowledge, learning process, and learning approach in human’s learning process in order. MAML is also a framework for training and design model, thus it has better versatility even in reinforcement learning [12,13,14].

A variety of MAML approaches have been proposed since its first introduction by Finn et al. [9]. Probability theories are often used in meta-learning to make better use of limited training data, a good example is using Bayesian model to establish a relationship between the posted results and the pre-trained data with Bayesian prior probability [15,16,17]. Entropy-base approaches such as Meta-SGD [18] and TAML [19] make meta-learners learn an unbiased initial model with the largest uncertainty over the output labels by preventing it from over-performing in classification tasks. Inspired by the Residual network (ResNet) [20] and Dense network (DenseNet) [21] in other machine learning applications, Qiao et al. [22] proposed the use of a deeper and more complex neural network in a meta-learner and reach a better performance in the feature extraction. As Arnold et al. [23] point out, MAML-based models need much more network parameters than theoretically needed. To reduce the number of network parameters required to be updated during training process, some works apply a pretrained operation on meta-learner’s feature extractor to generate a feature extractor with a better generalization performance [22,24,25].

Although MAML-based models can provide good performance in general, the existing models still have some problems, which hold back the methods from to going further.First of all, a meta-learner updates from a random initialization status, which forces the model to refresh all parameters of network every time. Secondly, a meta-learner performs an evaluation on every step in an adapting process but only considers the results of the last step as a loss value in judging the performance. Last but not least, the differential-based gradient decent which is often adopted in MAML models cannot guarantee the stability and accuracy in every gradient descent step, especially when there are limited training samples and updating steps. An overarching goal of this research is to overcome these problems and propose a more robust and accurate MAML-based meta-learning model.

### 2.2. Gradient Descent Optimization in Meta-Learning Models

Early research on optimizing a gradient descent by exploring the geometry of homogeneous space can trace back to decades. Such research treat networks as matrices in Manifold learning [26,27] and Lie group learning [28,29] and constrain matrices to orthogonal group. Recently, some researcher has applied these methods to meta-learning models to improve the stability and accuracy of the models in gradient decent [30]. Nishimori et al. [31] explored the gradient on high-dimension space, which allowed a meta-learner make better use of limited data and information in Euclidean space. The authors extend this theory to a more generic case by treating a linear layer as an orthogonal-constrained Stiefel manifold and use a Lie group to transform the calculation process into a linear approximation. The approach has been applied to an image classification problem in research by Yang et al. [32] and showed a significant improvement in the optimization step. Inspired by the idea of the method by Nishimori et al., the proposed method in this research constrains a network to Stiefel manifold so that a meta-learner could perform a more stable gradient descent in limited steps and accelerate the adapting process.

## 3. Methods

**Figure 1:**
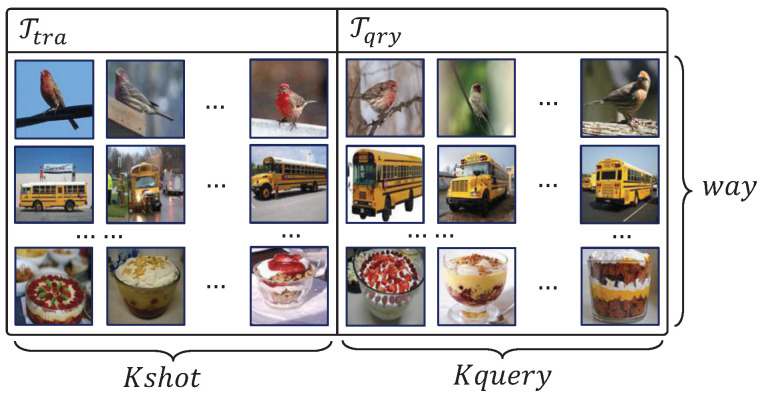
Overview of a task T used in meta-train phase includes $T_{tra}$ and $T_{qry}$. As described above, the number of $T_{tra}$ is Kshot and the number of $T_{qry}$ is Kquery.

A meta-learning model using several tasks {T}∼p(T) to train meta-learner in which T is the smallest cell of training data, also denoted as meta-task is used. The training batch contained multi tasks, each task consisting of a training set and query set, denoted as $T=\{T_{tra},T_{qry}\}$. The meta-learner updates the network parameters by using $T_{tra}$, then output the classify prediction of $T_{qry}$. The common setup for few-shot learning problem is 5-way 1-shot and 5-way 5-shot, meaning that each task consists of 5 classes data and the count of training samples is 1 or 5, the number of samples used to update model denoted as Kshot. The detailed diagram of T is shown in Figure 1. For example, a 5-way 1-shot learning problem means the meta-learner has been used to classify 5 classes with only one training sample. A full train-test process contains two parts, the *meta-train* and *meta-test*, the model training meta-learner in the *meta-train* phase, and then the use of datasets that have never been used to test the meta-learner’s performance of adapting to new unseen tasks.

**Figure 2:**
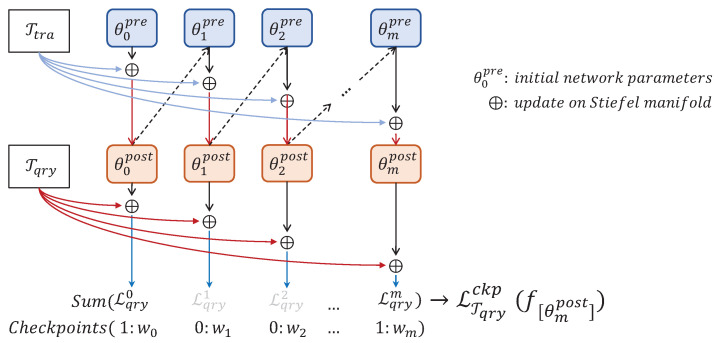
Diagram of our method, a full inner loop process to optimize network in meta-learner. Meta-learner make a prediction by $T_{tra}$, then update parameters of the network by compare the prediction with grand truth labels, and then generate outputs on $T_{qry}$ as the level of model’s learning performance.

The detail of the MAML algorithm is to embed the meta-learner, a fully functional neural network model into the meta-learning model, which is used to save and generate the initial parameters. The meta-learner can adapt to new unseen tasks T by fine tuning the network from a few samples that contain the same labels in T. After the meta-learner adapts to unseen task T, new samples are used to measure the performance of the meta-learner by accuracy, then the model use optimizer is used to optimize the loss function object to update parameters. MAML-based models constitute a dual-layer loop structure that allows the model to optimize the adapting process as a whole so as to equip the initial knowledge with a great generalization performance. A diagram of the inner loop process is shown in Figure 2, pre and post in Figure 2 represent the parameters in the network before and after adapting.

When the meta-learner finishes learning from $T_{tra}$, the model generate loss function based on the prediction of $T_{qry}$, then use the optimizers such as SGD or Adam [33] to optimize it. In this paper, we denoted the process by which meta-learner updates its parameters by $T_{tra}$ as inner loop, and denote the optimizing process on the meta-learning model as outer loop. Therefore, we conclude that the largest difference between the no-meta-learning structure model and MAML-based model is that the MAML-based model does not only optimize the performance of the meta-learner’s prediction accuracy but also make possible the optimization process as a whole, no matter what a meta-learner may do and how it did during inner loop. After every inner loop is finished, the model loss function based on $T_{tra}$ allows the model to use almost any approach to optimize the result from the inner loop process. The updating rules of the inner and outer loop in MAML is as follows, *k* in the following formula means the index of current task in the batch of tasks and *i* is the current step of inner loop.

(1) $$\theta_{k,i}=\theta_{k,i-1}-\alpha \nabla_{\theta_{k,i-1}}L_{T^{k}}f\left(\theta_{k,i-1}\right)$$

(2) $$\theta \leftarrow \theta -\beta \nabla_{\theta }\left(\sum_{T^{k}∼mini-batch}L_{T^{k}}\left(f\left(\theta_{k,m}\right)\right)\right).$$

### 3.1. Pretrained by Large Scale Data

A meta-learner needs large scale training data to equip generalization when facing unseen tasks, but it is extremely unstable when few samples are used to update the whole network. Thus, MAML and many MAML-based models tend to use a very shallow network that only contain 4 convolutional layers and a few fully connected layers to avoid a serious overfitting problem, but meta-learner requires a high accurately feature extraction from samples, as mentioned before, as it is almost impossible to update the whole network parameters at the same moment.

The training of meta-learner’s network parameters starting from random initialization in the meta-train phase, causes every single parameter in the meta-learner to require an update to fit few-shot setup conditions. The whole model should focus more on how to adapt under a series of limitations rather than learning how to extract features from samples. Recently, many meta-learning models split the meta-learner into two pieces logically: Feature extractor ϕ and the Classify layer θ, thus some models trend to pre-train the feature extractor part on large scale dataset [22,24,25]. Starting the meta-train phase after the pre-train process is completed can greatly reduce the pressure for the meta-learner.

Models in few-shot learning and some transfer learning approaches actually learn bias which is related to new tasks [34]. Our approach of pre-train relates to the work of Qiao et al. [22]. Firstly, we constructed a model with deep neural network trained on mini-ImageNet dataset’s subset [train∪val], then saved weights except for the classify layer. Additionally, these parameters ϕ were transferred to another model to classify the dataset’s test set and only then updated the classify layer to confirm the performance of this feature extractor. The updating rule is shown in Equation (3), fc here means the fully connected layer.

(3) $$\theta \leftarrow \theta -\alpha \nabla_{\theta_{fc}}Lf\left(\theta \right).$$

The conclusion drawn is that it is possible to extract features from other data with different classes using network trained on large scale dataset. The pretrain algorithm is summarized in Algorithm 1.

**Algorithm 1** Pretrain**Require:**D: {mini−batch}∼p(T) on large dataset
**Require:**
α,epoch: learning rate and number of training epochs 1:Randomly initialize feature extractor ϕ and classify layer θ2:Denote L as loss function3:**for**i<epoch**do**4: **while** not done **do**5:  Sample mini-batch $\left\{\left(x_{0},y_{0}\right),\left(x_{1},y_{1}\right)\ldots \right\}$ from D
6:  Optimize [ϕ, θ] by gradient descent: $\phi \leftarrow \phi -\alpha \nabla_{\phi }L_{mini-batch}\left(f_{\left[\phi ,\theta \right]}\right)$, $\theta \leftarrow \theta -\alpha \nabla_{\theta }L_{mini-batch}\left(f_{\left[\phi ,\theta \right]}\right)$7: **end while**
8:**end for**9:Save parameters ϕ as pretrain initial $\phi_{pre}$


The training feature extractor on different dataset could reduce the burden of the meta-learner during the adapting processes and improve the whole model performance significantly. We modified the updating methods both in the inner loop and outer loop. For the inner loop, the meta-learner only applied gradient descent on its classify layer and fixed other parameters, which can speed up the program and reduce space cost. For the outer loop, not every parameter was optimized, the model optimized the meta-learner’s classify layer, all bias, convolutions layers of which its filter’s kernel size is (1×1) and related batch normalization parameters. Specifically, our meta-learner used a wide residual network [35] with deep scale WRN-K for 10, selective optimization, reducing the number of parameters needed to be updated by 90.9% compared to method of MTL [25]. Our optimize principle trends to updating inductive biases of network to keep the ability of continuous adapting.

### 3.2. Multi-Stage Optimization

Meta-learning can be compared to a human’s growth process, for example the initialization parameters learned through the meta-train phase are similar to the structured general knowledge formed during the human learning process, and the method used in inner loop could be seen as the way a human learn new processes. Inspired by pedagogy, a long education process can be split into several stages and a human often test the performance at regular intervals to identify problems and make improvements on a timely basis rather than only focus on a final exam result.

The inner loop process is similar to human education, the increases of performance during the inner loop adapting process are not linear. The accuracy on $T_{qry}$ increased rapidly in the first half but almost stopped increasing in the middle even if there were still steps to end. Other MAML-based approaches only focused on what a meta-learner does in the last step of the inner loop [9,10,11] and they have not optimized the performance in time when the meta-learner enters the bottleneck, causing lots of hidden information to be wasted during adapting processes.

Under the premise of optimizing the loss function of the last step, we added some checkpoints ckps to match the inner loop’s bottleneck places. This ensures that the accuracy keep increasing to post the difficult points and keep the accuracy increasing until meets end. A meta-learner produces a copy $\left[\phi ,\theta^{{}^{\prime }}\right]$ of network parameters [ϕ,θ] at every step in the inner loop. A model uses these parameters to make predictions on $T_{qry}$, which is compared to the grand truth labels to generate loss function of this step. For the count of the inner loop denoted as *m*, we manually decided where it would be optimized. ckps are indexes which denote the place to joint current loss function into whole loss function, optimized in to the outer loop. ckps are as follows:

(4) $$ckp:=[r_{1},r_{2},\ldots ,r_{i}]\;\;i<m.$$

With step index in ckp, collecting loss function $L_{T_{qry}}\left(f_{\left[\phi ,\theta^{{}^{\prime }}\right]}\right)$ by using query set $T_{qry}$, model collected i+1 loss functions after inner loop completed: $L_{T_{qry}}^{ckps}\left(f_{\left[\phi ,\theta^{{}^{\prime }}\right]}\right)=\sum L_{T_{qry}}^{ckp_{i}}\left(f_{\left[\phi ,\theta^{{}^{\prime }}\right]}\right)$, simplify denoted as $L_{qry}$, then adjust their importance by multiplying the weight coefficient $W_{m}=\left[w_{1},w_{2},\cdots w_{i}\right],\;0<w_{x}<1$. The update approach of outer loop shows in following, where grad means the gradient function:

(5) $$\left[\phi ,\theta \right]\leftarrow \left[\phi ,\theta \right]-\beta \left(grad_{\left[\phi^{{}^{\prime }},\theta \right]}W_{m}⊗L_{qry}\right).$$

### 3.3. Lie Group Network Constrained

As mentioned before, meta-learning are required using as few steps as possible when adapting to novel tasks to satisfy the requirements of rapid adaptation.

Most meta-learning methods apply updating operation by gradient descent ∇f(θ), but it is difficult for the meta-learner to generate the gradient, which can along nearest line when network holds tons of parameters. The sensitivity of the Euclidean gradient descent (E-GD) to the initial value of the learning rate also makes these models violate the original intention of the design such as adaptability and robustness. Although there are proofs that the Euclidean space-based gradient descent offers a better performance than some other adaptive optimizers [36], the conditions of meta-learning limits the number of steps, reducing the upper limit of gradient descent.

Adaptive optimizers like Adam [33], AdaDelta [37], and SGD [38] usually have faster convergence speeds during the model training phase with the same size of steps. When the number of steps decreases further, the advantage over E-GD becomes tiny, which could be explained as adaptive optimizers usually change the length of each steps but not edit the direction of descent *V*. Model works on classifying the layer’s parameters θ in the inner loop process, the single fully connected linear layer structure allows us define θ as a matrix $w\in R_{m\times n}$, with a dimension of m×n, with elements in x∈R, and by setting m>n to simplify the expression.

Thus the output pass through feature extractor and classify layer of meta-learner can be modeled as:

(6) $$output=softmax(w^{T}x+w_{bias}).$$

Updating rule by E-GD to optimize the loss function formed by outputs and grand truth labels as follows, α is the step size:

(7) $$w\leftarrow w-\alpha \nabla_{w}L_{x}f\left(w\right).$$

The network’s parameter search space is $R_{m\times n}$ when using E-GD, so it is difficult for the model with few updates reach the convergence in time. There are perhaps some latent geometric structures among neural networks [31] and this allows us to treat the fully connected linear layer as a manifold. Gradient descents on the manifold have some advantages to E-GD: (i) Manifold limits by conditions, this significantly reduce the scale of parameter space and (ii) the gradients via geodesic flows offer a better accuracy to speed up convergence process. The geodesics is a distance-minimizing curve between points in space with or without structures. The optimizing process of the neural network can be seen as connecting the initial value position and stationary point of the loss function with the shortest path. The orthogonal constraint often be used when considering add conditions to parameter matrix *w*:

(8) $$w\in St\left(m,n\right)=\left\{w\in R_{m\times n}|w^{T}w=I_{n}\right\}.$$

This constraint reduces the scale of parameter search space during gradient descent and improve the accuracy of direction. Matrices satisfy this constraint are the points in the Stiefel manifold, which can be seen as a Lie group, meaning all *w* of networks updating process constitute the Stiefel manifold that belong to a O(m,n) homogeneous space as the subspace of orthogonal Lie group O(m). Denote St(m,n) as a Stiefel manifold with dimension m×n, O(m,n) an orthogonal space with dimension m×n and the dimension of O(m) is m×m. The regular way of calculating geodesic on the manifold is searching nearest the subset from start point to target position [39,40].

Firstly, generate the neighborhood subset $\cup_{s}=\left\{x_{i}|0<i<l\right\}$ of start point $x_{s}$ and analyze this subset by proposing an invariant scalar product $g_{ij}\left(x_{i}\right)$ under the automorphism at the identity element position, then calculate $g_{ij}\left(x_{i}\right)$ and use results into the equation as follows:

(9) $$d\left(x_{i},x_{j}\right)=\sqrt{\sum_{n}^{i,j=1}g_{ij}\left(x_{i}\right)\left(x_{ii}-x_{ji}\right)\left(x_{ij}-x_{jj}\right)}.$$

Find every $x_{i}$ which is less than the threshold *d* by calculating the Euclidean distance between $x_{i}$ and $x_{j}$ in set $\cup_{s}=\left\{x_{i}|0<i<l\right\}$. Repeat this process until the geodesic from start point $x_{s}$ to end $x_{e}$ comes up.

But this approach costs too much computing resource because every point search paths passed by has its neighborhood, these neighborhoods contain some elements which also have their own neighborhoods and this defect becomes more and more serious as the dimension of parameter space increases. St(m,n) is usually embedded into a O(m) matrix: $\tilde{W}=\left(w,I_{(}m,m-n\right))$ by adding O(n−p) orthogonal column vectors, $\tilde{W}$ is a special case of Stiefel manifold when m=n. So it is possible to use the Lie algebra to simplify the geodesic searching process on an orthogonal group. Linear mapping based on matrix multiplication could naturally project O(m) to St(m,n). Stiefel manifold itself is a homogeneous space, thus there is transitive action by the Lie group on it. From the initial point ${\tilde{W}}_{0}$, the network value can reach every position in Stiefel manifold by left multiplication G-action ${\tilde{W}}_{1}=R{\tilde{W}}_{0}$ [28].

Denote $G\left(\tilde{W}\right)=\left\{W\in St\left(m,n\right)|W=RW,R\in O\left(m\right)\right\}$ as the space $R{\tilde{W}}_{0}$ that could be reached, its equivalent to the parameter space with orthogonal constraint. So the method which update the parameter matrix by multiplication O(m) is feasible. Nature gradient via geodesic flow on O(m) can be obtained by the Lie algebra exponential map, which is proposed by Yasunori [31], where *g* represents the projection function from O(m) to St(m,n).

(10) $$\tilde{W}\leftarrow \tilde{W}exp\left(-\gamma {\tilde{W}}_{n}^{t}{\tilde{V}}_{H}\right)$$

(11) $${\tilde{V}}_{H}=\lambda \left\{\nabla f\left(g\left(\tilde{W}\right)\right)-\tilde{W}\nabla f{\left(g\left(\tilde{W}\right)\right)}^{T}\tilde{W}\right\}$$

(12) $$g\left(\tilde{W}\right):O\left(m\right)\to St\left(m,n\right),\tilde{W}\in O\left(p\right),g\left(\tilde{W}\right)=w\in St\left(m,n\right).$$

The linear approximation could avoid exponential calculations by projection gradient from O(m) in $\tilde{W}$ to St(m,n) [41]. Natural gradient on the Stiefel manifold described by the following formula, $grad_{st}$ means the gradient under the constraint of the Stiefel manifold:

(13) $$grad_{St}\left(f\left(w\right)\right)=\nabla f\left(w\right)-w\nabla f{\left(w\right)}^{T}w.$$

Our method replaces the differential in the Euclidean space with natural gradient in the Stiefel manifold and comes up with the updating rule in the inner loop process:

(14) $$\theta^{{}^{\prime }}=\theta -\alpha \left(\nabla_{\theta }L_{T_{tra}}\left(f_{\left[\phi ,\theta \right]}\right)-\theta \nabla_{\theta }^{T}L_{T_{tra}}\left(f_{\left[\phi ,\theta \right]}\right)\theta \right).$$

We also compared the performance of the model using Stiefel manifold structure and E-GD under the same other conditions. The method that uses linear approximation natural gradient descent guarantees the computational efficiency in backpropagation, and does not introduce new extra parameters. Approaches in meta-train phase are presented in Algorithm 2.

**Algorithm 2** Meta-Train**Require:**$\phi_{pre}$: pretrained feature extractor parameters **Require:**{α},β,γ: learning rates for inner loop and outer loop **Require:**p(T): tasks from training data
1:Restore values from $\phi_{pre}$ to ϕ2:Initialize classify layer θ to orthogonal matrix as a Stiefel manifold3:**while** not done **do**4: Sample tasks from p(T) as a batch5: **for all** $\left\{T^{i}\right\}$ in tasks **do**6:  **Algorithm 3**7: **end for**8: Sum loss functions of evaluation results in every checkpoint in **Algorithm 3**: $L_{qry}=\sum L_{T_{qry}}\left(f_{\left[\phi ,\theta^{{}^{\prime }}\right]}\right)$
9: Optimize $\phi \leftarrow \phi -\beta \left(\nabla_{\phi }L_{qry})\right)$ by optimizer 10: Update $\theta \leftarrow \theta -\gamma \left(\nabla_{\theta }L_{qry}-\theta L_{qry}^{T}\theta \right)$ via geodesic flows on the Stiefel manifold11:**end while**

**Algorithm 3** Inner Loop**Require:**T: a task in current batch **Require:**{α}: learning rates of gradient descent
1:Split T into $\left\{T_{tra},T_{qry}\right\}$2:Get Euclidean gradient: $\nabla_{\theta }L_{T_{tra}}\left(f_{\left[\phi ,\theta \right]}\right)$ using $T_{tra}$, denoted as $grad_{tra}$3:Update θ by natural gradient descent on Stiefel manifold with scaling {α}: $\theta^{{}^{\prime }}=\theta -\left\{\alpha \right\}⊗\left(grad_{tra}-\theta grad_{tra}^{T}\theta \right)$
4:Evaluate $\theta^{{}^{\prime }}$ on $T_{qry}$ and get loss function: $L_{T_{qry}}\left(f_{\left[\phi ,\theta^{{}^{\prime }}\right]}\right)$ when reach checkpoints

## 4. Experiments

### 4.1. Dataset

The dataset we used in this experiment is a universal image dataset for few-shot learning: Mini-ImageNet [4], which is a subset of ImageNet proposed by Deng et al. [42]. Mini-ImageNet includes 100 image classes, each of which has 600 natural photos that are cropped to 80×80 pixels in meta-learning models. In this paper, this dataset is divided into three sets: “train” (64 classes), “val” (16 classes), and “test” (20 classes).

### 4.2. Model Detail

The process of the experiment consists of three stages: Pretrain, meta-train, and meta-test. The “train set” and the “val set” are used during pretrain and meta-train phases while the “test set” is only used in the meta-test stage. In addition, the meta-learner shares the same feature extractor structure in every phase.

#### 4.2.1. Pretrain Phase

We train a model on the subset [train∪val] of the mini-ImageNet to classify 80 image classes and generate a feature extractor. The network used in the feature extractor is the wide residual network [35] with 22 convolutional layers extended from a standard Residual network [20]. The depth scale of filters in convolutional kernels is 10, so this network is denoted as WRN-22-10.

The input data is preprocessed by the initial part of network which contains Conv-Batch normalization-ReLU to reduce the size of raw images. The depths of filters in convolutional kernels increased in the beginning of every residual block and corresponded to the schedules [160,320,640]. After the initial process, there were residual blocks that contained a few convolutional layers and some activation functions, often repeated for times to extend the depth and layers of network. Convolutional layers with a 3×3 filter kernel shrank the size of feature map with 2 stride and increased the dimension of depth, then operations Batchnormalization–ReLU were applied before the data met the second convolution layer. In the last part of a residual block, we added the processed feature map to the original input data, which is called shortcut after two convolutional operations.

Specifically, we inserted a dropout layer between convolutional layers in each residual block to change the structure to wide-dropout, which is the type of convolutional layers we used, B(3,3). According to Zagoruyko et al. [35], a dropout layer inside the block could prevent overfitting. The *keep-rate* of these dropouts was set at 0.5 to max the generalization performance. After 9 residual blocks, a global average pooling layer was applied before the following fully connected layers, which included two linear layers with ReLU as the activation function to further reduce the size of feature map and flatten it. Finally, we used the SGD optimizer to the pretrain model with the base learning rate which was 0.1 to update network parameters and set the learning rate decays by 20% per 30 epochs.

#### 4.2.2. Meta Train Phase

We used a single linear layer as the classify layer because the updating rule changed to natural gradient via geodesic. The structure of the feature extractor in meta-train phase was the same as that of the pretrain model. Moreover, the parameters of the feature extractor were extended from the pretrained model. We used the wide-dropout [35] inside residual blocks, which was disabled in the meta-test stage. The parameters were split into two parts during meta-train and meta-test: Classify layer θ and trained feature extractor $\phi^{{}^{\prime }}$. A strategy “the initial learning rate multiply 0.99 after every gradient descent operation” was also applied in the inner loop. After defining the structure and parameters of our model, the optimizer Adam [33] with an initial learning rate 1 × 10^−4^ was applied to update the bias matrix of feature extractor. Due to the orthogonal structure of the classify layer, the last linear layer was updated by the Stiefel manifold natural gradient with a fixed learning rate 0.01 to keep the constraint. In the meta-train phase, the pressure of the model was significantly reduced since the feature extractor was trained in the pretrain phase, which gave the proposed method an advantage over other MAML [9] models on the requirement of data. The model iterated for 15,000 epochs and required 5000 tasks.

## 5. Results

**Figure 3:**
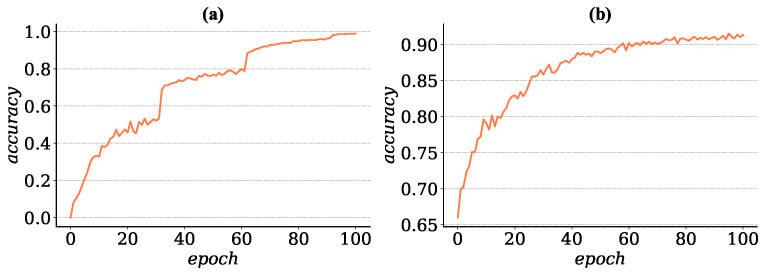
(**a**) Testing accuracy on mini-ImageNet dataset during pretrain phases for 100 epochs. (**b**) The accuracy that transfer feature extractor to classify test subset, result shows that the model could reach a high accuracy with only trains the classify layer.

We performed a pretrain operation on the feature extractor of meta-learner and recorded the corresponding increase of accuracy with training steps (see Figure 3a). As seen from the figure, the WRN-22-10 could handle an 80-way image classification problem, the prediction accuracy increased greatly with the decrease of the learning rate in each stage, and the test accuracy reached 99% after 100 training epochs and 3 stages of learning decay. We also tested the generalization performance of the feature extractor by replacing the 80-way classification model’s last layer with a 16-way fully connected linear layer and tried to adapt the new model to test the subset with the updating rules that only the classify layers that were optimized. As shown in Figure 3b, the feature extractor $\phi^{pre}$ which is trained on a large-scale data could achieve a high accuracy on the “test set”.

**Figure 4:**
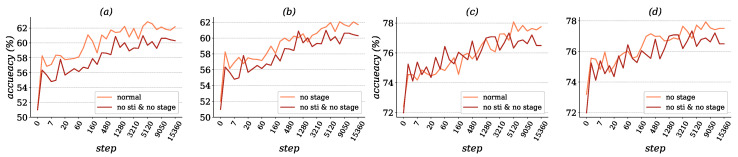
(**a**,**b**) Represent the accuracy results of 5-way 1-shot learning on mini-ImageNet dataset with different settings, **no sti** in figures means gradient descent is Euclidean gradient, **no stage** is the setting that disable the multi-stage optimizing method. (**c**,**d**) show results under the setting 5-way 5-shot.

The evaluation results of the 5-way 1-shot and 5-shot tasks in the meta-train process are presented in Figure 4. It is important to tell the contributions of each module of our methods. Results show that a deeper network provider significantly made improvements on the feature extracting performance, the accuracy remained low if the pretrain was not applied, so only two other methods were used for comparison. Replacing Euclidean-based gradient descent with natural gradient descent in the Stiefel manifold provided significance improvement in both 1-shot and 5-shot learning with almost 1.3% and 2.19% respectively. The application of multi-stage checkpoints made the a smoother improvement of accuracy, although the testing of accuracy gradual convergence took a bit more time (about 5000 training steps) for the model to reach the best performance under the condition of 5-shot tasks.

In Table 1, the classification accuracy of our methods (referred to as Multi-Stage Meta-Learning (MSML) in the table) is presented. As shown in the table, MSML gained a high accuracy performance in the 5-way 1-shot learning problem for the image dataset mini-ImageNet. To measure the performance of each module of our methods, their contributions are also reported in Table 1. MSML received a relatively high accuracy without multi-stage optimization and natural gradient-based updating rule, which were 60.8±0.87% for 5-way 1-shot tasks and 76.13±0.66% for 5-shot tasks. As shown in Table 1, the application of MSML added a significant improvement in the accuracy with the multi-stage optimization costing more memory during the training process.From the result in the last row in Table 1, the testing accuracy of MSML reached 62.42±0.76% in 1-shot problem and 77.32±0.66% in 5-shot tasks.

## 6. Discussion

As mentioned above, we saved the network after the pretrain phase and restored parameters in the feature extractor to a 16-way image classification neural network and froze them. As found in Figure 3b, the testing accuracy reached about 93% by only optimizing the last layer of the model, which indicates that it is practicable for the meta-learner to gain an excellent performance by updating a few parts of network instead of refreshing every parameter like MAML [9] and Reptile [10].

After proving the possibility of training a well-generalization feature extractor to handle unseen classes, experiments were performed on the influence of the updated parameters’ scale on the accuracy. As shown in Figure 4, our model achieved a similar accuracy level as that of MTL [25] but used far less parameters for model updating. At this stage, it could be concluded that the performance of classifying a layer plays a key role in few-shot problems. Compared with the thousands updates during the pretrain and transfer learning, only a few updating steps were available to classifying a layer during the meta-train phase, which reduces the performance of the meta-learner.

In Table 2, the classification accuracy of our method (referred to as MSML in the table) is compared to those of other methods (e.g., Matching Nets [4], MTL [25], and TAML [19]). MSML achieved the best performance in the 5-way 1-shot tasks and with a similar result as that of LEO [24] in 5-shot tasks. Compared with MTL, MSML demonstrated a significant improvement by using manifold learning approaches and multi-stage optimization, which enhanced the accuracy by 1.22% and 1.82% in 1-shot and 5-shot tasks respectively in comparison with those of MTL. The advantage of MSML in the accuracy over other methods was based on MSML using less tasks: 5000 for MSML, 8000 for MTL, and 24,000 for MAML. Based on the comparison in Table 2, we could draw a conclusion that metric-based models have encountered a serious bottleneck in dealing with high dimension data such as real world images [5,6], and that gradient-based methods show a great potential in their compatibility with complex networks.

## 7. Conclusions

In this paper, we proposed a novel meta-learning model using multi-stage joint training approach. By saving intermediate states during adapting process, the proposed method postponed the bottleneck position to increase the performance of meta-learner. Moreover, constraining the network to a Stiefel manifold also offered a better descent accuracy. An experiment on the mini-ImageNet dataset demonstrated that the proposed method reached a better accuracy under 5-way 1-shot and 5-way 5-shot conditions.

Despite the initial success, our model at the current stage had two problems. For one thing, it still could not automatically improve the existing feature extractor when facing a new dataset, which limited the upper bound of the model performance, even training and testing on the same dataset. For another, the optimization process used different calculation rules and corresponding learning rates for the two logical parts of the model, which resulted in a coordination problem between the two parts of the model through the adaptive learning rates. For the future development, we plan to explore an approach for harmonizing the step size of Euclidean and manifold-based gradient. In addition, measuring the impact of different classify layers that design on the overall efficiency of meta-learning models and developing a premium updating rule of classify layers are two possible research directions.

## 8. Materials and Methods

### 8.1. Computational Requirements

Code used in experiments were based on PyTorch and runs on Linux. Softwares required are Python 3.6, CUDA, and cuDNN. We recorded the resource usage when running these experiments, the program requires 10.1 GB GPU memory and 1.8 GB memory under the setup of 5-way 5-shot learning.

### 8.2. Program Availability

Dataset and runnable code used in our experiments are available in https://github.com/Chacrk/MSML_Project, detailed running instructions is also available in repository.

### 8.3. Running Experiment

Download mini-ImageNet dataset, images are cropped to (84×84), unzip file by run command *python proc_dataset.py* in the data folder. Enter directory *pretrain* and run *python pretrain.py* to get pretrained weights data.

Pretrain phase last about 5.1 h by using NVIDIA TITAN Xp and Intel Xeon E5-2620 v4. Enter meta folder while pretrain process done and run command *python main.py* to run experiment.

## Figures and Tables

**Table 1:** Image classification accuracy on mini-ImageNet with different methods.

Methods	1-Shot	5-Shot	Memory Cost (MB)	
			1-Shot	5-Shot
**MSML without Sti and Stage**	**60.80** ± **0.87%**	**76.23** ± **0.66%**	4062	6443
**MSML without Sti**	**61.12** ± **1.02%**	**76.19** ± **0.41%**	8655	9984
**MSML without Stage**	**61.50** ± **0.82%**	**77.02** ± **0.73%**	4165	6521
**MSML**	**62.42** ± **0.76%**	**77.32** ± **0.66%**	8767	10,157

**Table 2:** Image classification results on mini-ImageNet under the setting of 5-way 1-shot or 5-way 5-shot. Results in this table reported from their papers.

Few-Shot Learning Method	1-Shot	5-Shot
**Metric Based**
Matching Nets [4]	43.56±0.84%	55.31±0.73%
Relation Nets [5]	50.44±0.82%	65.32±0.70%
Prototypical Nets [6]	49.42±0.78%	68.20±0.66%
**Gradient descent based**
MAML (4 Conv) [9]	48.70±1.84%	63.11±0.92%
Reptile (4 Conv) [10]	49.97±0.32%	65.99±0.58%
meta-SGD (4 Conv) [11]	50.47±1.87%	64.03±0.94%
LEO (WRN-28-10) [24]	61.76±0.15%	77.46±0.12%
MTL (ResNet-12) [25]	61.20±1.80%	75.50±0.80%
TAML (4 Conv) [19]	51.73±1.88%	66.05±0.85%
**MSML (The proposed method)**	**62.42** ± **0.76%**	**77.32** ± **0.66%**
**Memory augmented based**
TADAM [7]	58.50±0.30%	76.70±0.30%
SNAIL [8]	55.71±0.99%	68.66±0.92%

## References

[1] Szegedy C., Vanhoucke V., Ioffe S., Shlens J., Wojna Z. Rethinking the inception architecture for computer vision. Proceedings of the IEEE Conference on Computer Vision and Pattern Recognition.

[2] Glass G.V. (1976). Primary, secondary, and meta-analysis of research. Educ. Res..

[3] Powell G. (1980). A Meta-Analysis of the Effects of “Imposed” and “Induced” Imagery Upon Word Recall. https://eric.ed.gov/?id=ED199644.

[4] Vinyals O., Blundell C., Lillicrap T., Wierstra D. Matching networks for one shot learning. Proceedings of the 30th Conference on Neural Information Processing Systems (NIPS 2016).

[5] Sung F., Yang Y., Zhang L., Xiang T., Torr P.H., Hospedales T.M. Learning to compare: Relation network for few-shot learning. Proceedings of the IEEE Conference on Computer Vision and Pattern Recognition.

[6] Snell J., Swersky K., Zemel R. Prototypical networks for few-shot learning. Proceedings of the 31st Conference on Neural Information Processing Systems (NIPS 2017).

[7] Oreshkin B., López P.R., Lacoste A. Tadam: Task dependent adaptive metric for improved few-shot learning. Proceedings of the 32nd Conference on Neural Information Processing Systems (NeurIPS 2018).

[8] Mishra N., Rohaninejad M., Chen X., Abbeel P. (2017). A simple neural attentive meta-learner. arXiv.

[9] Finn C., Abbeel P., Levine S. Model-agnostic meta-learning for fast adaptation of deep networks. Proceedings of the 34th International Conference on Machine Learning.

[10] Nichol A., Achiam J., Schulman J. (2018). On first-order meta-learning algorithms. arXiv.

[11] Li Z., Zhou F., Chen F., Li H. (2017). Meta-SGD: Learning to learn quickly for few-shot learning. arXiv.

[12] Gupta A., Eysenbach B., Finn C., Levine S. (2018). Unsupervised meta-learning for reinforcement learning. arXiv.

[13] Nagabandi A., Clavera I., Liu S., Fearing R.S., Abbeel P., Levine S., Finn C. (2018). Learning to adapt in dynamic, real-world environments through meta-reinforcement learning. arXiv.

[14] Al-Shedivat M., Bansal T., Burda Y., Sutskever I., Mordatch I., Abbeel P. (2017). Continuous adaptation via meta-learning in nonstationary and competitive environments. arXiv.

[15] Kim T., Yoon J., Dia O., Kim S., Bengio Y., Ahn S. (2018). Bayesian model-agnostic meta-learning. arXiv.

[16] Yoon J., Kim T., Dia O., Kim S., Bengio Y., Ahn S. Bayesian model-agnostic meta-learning. Proceedings of the 32nd Conference on Neural Information Processing Systems (NeurIPS 2018).

[17] Finn C., Xu K., Levine S. Probabilistic model-agnostic meta-learning. Proceedings of the 32nd Conference on Neural Information Processing Systems (NeurIPS 2018).

[18] Ravi S., Larochelle H. (2016). Optimization as a Model for Few-Shot Learning. https://openreview.net/forum?id=rJY0-Kcll.

[19] Jamal M.A., Qi G.J. Task Agnostic Meta-Learning for Few-Shot Learning. Proceedings of the IEEE Conference on Computer Vision and Pattern Recognition (CVPR).

[20] He K., Zhang X., Ren S., Sun J. Deep residual learning for image recognition. Proceedings of the IEEE Conference on Computer Vision and Pattern Recognition (CVPR).

[21] Zhang Y., Tian Y., Kong Y., Zhong B., Fu Y. Residual dense network for image super-resolution. Proceedings of the IEEE Conference on Computer Vision and Pattern Recognition (CVPR).

[22] Qiao S., Liu C., Shen W., Yuille A.L. Few-shot image recognition by predicting parameters from activations. Proceedings of the IEEE Conference on Computer Vision and Pattern Recognition (CVPR).

[23] Arnold S.M., Iqbal S., Sha F. (2019). Decoupling Adaptation from Modeling with Meta-Optimizers for Meta Learning. arXiv.

[24] Rusu A.A., Rao D., Sygnowski J., Vinyals O., Pascanu R., Osindero S., Hadsell R. (2018). Meta-learning with latent embedding optimization. arXiv.

[25] Sun Q., Liu Y., Chua T.S., Schiele B. Meta-transfer learning for few-shot learning. Proceedings of the IEEE Conference on Computer Vision and Pattern Recognition (CVPR).

[26] Cai D., He X., Han J., Zhang H.J. (2006). Orthogonal laplacianfaces for face recognition. IEEE Trans. Image Process..

[27] Zhang Z., Chow T.W., Zhao M. (2012). M-Isomap: Orthogonal constrained marginal isomap for nonlinear dimensionality reduction. IEEE Trans. Cybern..

[28] Fan-Zhang L., Huan X. (2007). SO(3) Classifier of Lie Group Machine Learning. http://citeseerx.ist.psu.edu/viewdoc/summary?doi=10.1.1.114.4200.

[29] Li F.Z., Xu H. (2007). SO(3) classifier of Lie group machine learning. Learning.

[30] Huang L., Liu X., Lang B., Yu A.W., Wang Y., Li B. Orthogo nal weight normalization: Solution to optimization over multiple dependent stiefel manifolds in deep neural networks. Proceedings of the Thirty-Second AAAI Conference on Artificial Intelligence.

[31] Nishimori Y. (2004). A Neural Stiefel Learning based on Geodesics Revisited. WSEAS Trans. Syst..

[32] Yang M., Li F., Zhang L., Zhang Z. (2018). Lie group impression for deep learning. Inf. Process. Lett..

[33] Kingma D.P., Ba J. (2014). Adam: A method for stochastic optimization. arXiv.

[34] Battaglia P.W., Hamrick J.B., Bapst V., Sanchez-Gonzalez A., Zambaldi V., Malinowski M., Tacchetti A., Raposo D., Santoro A., Faulkner R. (2018). Relational inductive biases, deep learning, and graph networks. arXiv.

[35] Zagoruyko S., Komodakis N. (2016). Wide residual networks. arXiv.

[36] Keskar N.S., Socher R. (2017). Improving generalization performance by switching from adam to sgd. arXiv.

[37] Zeiler M.D. (2012). ADADELTA: An adaptive learning rate method. arXiv.

[38] Bottou L. (2010). Large-scale machine learning with stochastic gradient descent. Proceedings of COMPSTAT’2010.

[39] Belkin M., Niyogi P. (2004). Semi-supervised learning on Riemannian manifolds. Mach. Learn..

[40] Li F. (2013). Lie Group Machine Learning.

[41] Nishimori Y., Akaho S. (2005). Learning algorithms utilizing quasi-geodesic flows on the Stiefel manifold. Neurocomputing.

[42] Deng J., Dong W., Socher R., Li L.J., Li K., Fei-Fei L. Imagenet: A large-scale hierarchical image database. In Proceeding of the 2009 IEEE conference on computer vision and pattern recognition.

